# VIRMA-Dependent N6-Methyladenosine Modifications Regulate the Expression of Long Non-Coding RNAs CCAT1 and CCAT2 in Prostate Cancer

**DOI:** 10.3390/cancers12040771

**Published:** 2020-03-25

**Authors:** Daniela Barros-Silva, João Lobo, Catarina Guimarães-Teixeira, Isa Carneiro, Jorge Oliveira, Elena S. Martens-Uzunova, Rui Henrique, Carmen Jerónimo

**Affiliations:** 1 Cancer Biology and Epigenetics Group, Research Center of Portuguese Oncology Institute of Porto (GEBC CI-IPOP) and Porto Comprehensive Cancer Center (P.CCC), R. Dr. António Bernardino de Almeida, 4200072 Porto, Portugal; daniela.silva@ipoporto.min-saude.pt (D.B.-S.); jpedro.lobo@ipoporto.min-saude.pt (J.L.); catarina.guimaraes.teixeira@ipoporto.min-saude.pt (C.G.-T.); isa.carneiro@ipoporto.min-saude.pt (I.C.); henrique@ipoporto.min-saude.pt (R.H.); 2 Department of Pathology, Portuguese Oncology Institute of Porto (IPOP), R. Dr. António Bernardino de Almeida, 4200-072 Porto, Portugal; 3 Department of Urology, Portuguese Oncology Institute of Porto (IPOP), R. Dr. António Bernardino de Almeida, 4200-072 Porto, Portugal; jorge.oliveira@ipoporto.min-saude.pt; 4 Department of Urology, Cancer Institute, Erasmus MC University Medical Center Rotterdam, Be-432A, PO Box 2040, 3000 CA Rotterdam, The Netherlands; e.martens@erasmusmc.nl; 5 Department of Pathology and Molecular Immunology, Institute of Biomedical Sciences Abel Salazar, University of Porto (ICBAS-UP), Rua Jorge Viterbo Ferreira 228, 4050-513 Porto, Portugal

**Keywords:** prostate cancer, epitranscriptome, N6-Methyladenosine, VIRMA, long non-coding RNAs

## Abstract

RNA methylation at position N6 in adenosine (m^6^A) and its associated methyltransferase complex (MTC) are involved in tumorigenesis. We aimed to explore m^6^A biological function for long non-coding RNAs (lncRNAs) in prostate cancer (PCa) and its clinical significance. m^6^A and MTC levels in PCa cells were characterized by ELISA and western blot. Putative m^6^A-regulated lncRNAs were identified and validated by lncRNA profiler qPCR array and bioinformatics analysis, followed by m^6^A/RNA co-immunoprecipitation. Impact of m^6^A depletion on RNA stability was assessed by Actinomycin D assay. The association of m^6^A-levels with PCa prognosis was examined in clinical samples. Higher m^6^A-levels and VIRMA overexpression were detected in metastatic castration-resistant PCa (mCRPC) cells (*p* < 0.05). VIRMA knockdown in PC-3 cells significantly decreased m^6^A-levels (*p* = 0.0317), attenuated malignant phenotype and suppressed the expression of oncogenic lncRNAs CCAT1 and CCAT2 (*p* < 0.00001). VIRMA depletion and m^6^A reduction decreased the stability and abundance of CCAT1/2 transcripts. Higher expression of VIRMA, CCAT1, and CCAT2 as a group variable was an independent predictor of poor prognosis (HR = 9.083, CI95% 1.911–43.183, *p* = 0.006). VIRMA is a critical factor sustaining m^6^A-levels in PCa cells. VIRMA downregulation attenuates the aggressive phenotype of PCa by overall reduction of m^6^A-levels decreasing stability and abundance of oncogenic lncRNAs.

## 1. Introduction

Prostate cancer (PCa) is a highly prevalent malignancy, being the second most common cancer and the fifth leading cause of cancer-related death in men [1]. First line treatment of advanced PCa is androgen-deprivation therapy. Nevertheless, a proportion of patients develops resistance (Castration-Resistant Prostate Cancer, CRPC) and progresses to lethal metastatic disease (mCRPC). The molecular mechanisms underlying mCRPC are not yet fully understood. Hence, the discovery of new key players involved in PCa aggressiveness and CRPC phenotype are mandatory. 

RNA activity in cells is tightly regulated by different chemical modifications that can be dynamic and adaptable [2]. RNA methylation at position N6 in adenosine (m^6^A) is a highly prevalent epitranscriptomic modification in messenger and non-coding RNAs (ncRNAs) that affects splicing, translation, and stability [3]. Alterations in m^6^A are implicated in several biological processes in eukaryotes [4]. The m^6^A modification of RNA is dynamically regulated by methyltransferases (“writers”), binding proteins (“readers”), and demethylases (“erasers”) [5]. M^6^A is installed by the methyltransferase complex (MTC), a multi-component protein complex including a heterodimer of two methyltransferase-like proteins, METTL3 and METTL14, the Wilms’ tumor 1-associating protein (WTAP), and the Vir-like m^6^A methyltransferase associated protein (VIRMA). METTL3 is the only subunit of the complex that has catalytic activity [6]. METTL14 is important for substrate recognition, activity and specificity, while WTAP and VIRMA function as regulatory components. WTAP stabilizes the heterodimer formed by METTL3/14, plays a critical role for the nuclear localization of MTC, and can affect RNA alternative splicing. It has been proposed that VIRMA is important for the deposition of m^6^A at 3′UTR of messenger RNA [7]. VIRMA is required for the full methylation program in mammals and its silencing causes a substantial reduction in m^6^A levels during embryonic development [8]. Eraser/demethylases, such as FTO and ALKBH5, have a crucial role in the dynamics and control of m^6^A modification in target RNAs. Additionally, specific reader proteins like YTHDF1/2/3, eIF3, IGF2BP1/2/3, and HNRNPA2B1 can bind to the m^6^A consensus motif (DRACH), directly or indirectly affecting RNA stability and function [5]. The functional analysis of writer, reader and eraser proteins that modulate RNA modifications is of major importance to further unveil their biological significance.

The recent development of new high-throughput sequencing techniques brought new insights into our understanding of the transcriptome versatility and revealed that epitranscriptome homeostasis is disrupted in cancer. Emerging evidence demonstrates the importance of m^6^A and MTC in several human cancers [9]. For example, reduction of m^6^A-levels promotes tumorigenesis in glioblastoma stem cells [10], whereas METTL3 [11] and FTO [12] are associated with therapeutic resistance in pancreatic and cervical squamous cell carcinoma, respectively, constituting putative predictive biomarkers. Nevertheless, the full extent of functionality and implication of RNA modifications in cancer initiation and progression remains largely unknown. 

Long non-coding RNAs (lncRNAs) recently emerged as important players in prostate cancer biology. M^6^A is considered one of the most prevalent modifications in lncRNAs [13,14] and the characterization of lncRNA modifications occurring in PCa cells may disclose whether they affect key PCa signaling pathways [15]. 

To our knowledge, the m^6^A regulatory network underlying PCa onset and progression and its biological relevance are still largely unknown. In this study, we investigated the functions and specific mechanisms of m^6^A dynamics in the development of PCa aggressive phenotypes. VIRMA was found to play a pivotal role in establishment of m^6^A mark impacting also on PCa aggressiveness profile. Downregulation of screened m^6^A targeted-lncRNA was observed after VIRMA knockdown. Combination of VIRMA and lncRNAs expression independently predicted poor prognosis in PCa patients. Thus, we postulate a novel regulatory mechanism in which VIRMA knockdown decreases oncogenic lncRNA stability through reduction of m^6^A mark, attenuating the malignant features of PCa.

## 2. Results

### 2.1. In Silico Analysis of Methyltransferase Complex Expression in Prostate Adenocarcinoma

**Figure 1:**
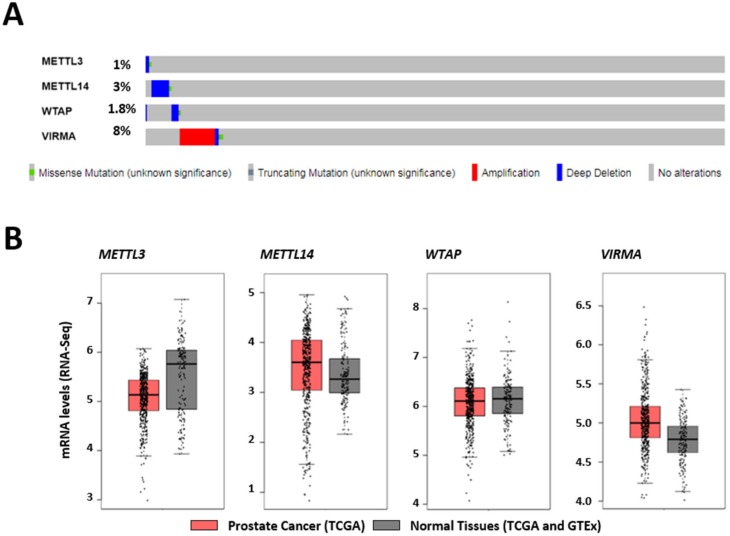

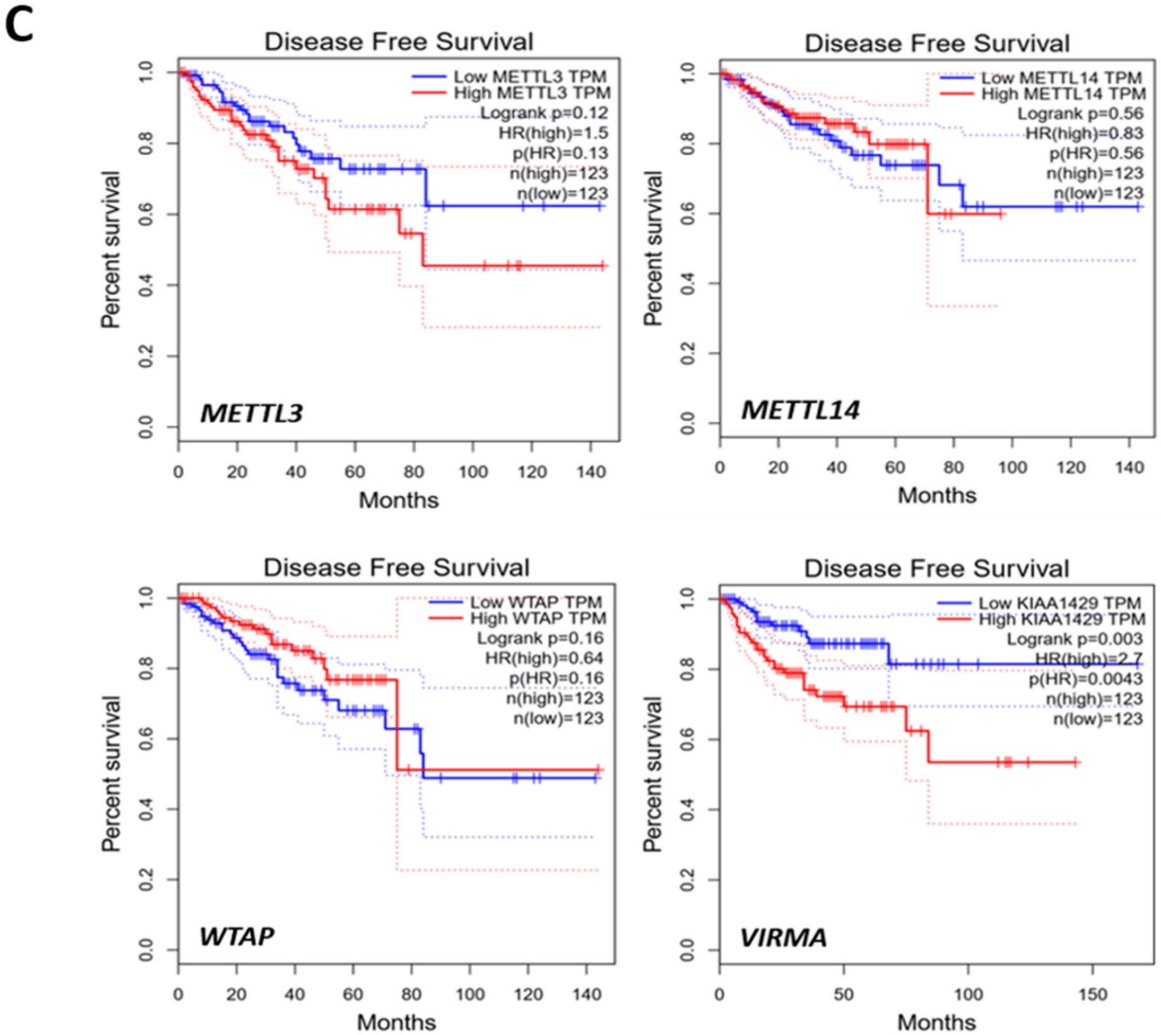
VIRMA expression is increased in prostate cancer (PCa) and associates with tumor relapse. (**A**) Alteration frequency of METTL3, METTL14, WTAP, and VIRMA in PCa TCGA cohort (*n* = 492). (**B**) mRNA expression levels of m^6^A modifying enzymes in clinical tissue samples of *The Cancer Genome Atlas* (TCGA) and GTEx prostate adenocarcinoma (*n* = 492, red boxplot) and adjacent normal specimens (*n* = 152, grey boxplot). (**C**) Disease-free survival curves of patients with high mRNA expression (red line) and with low mRNA expression (blue line) (Mantel–Cox test).

To interrogate the biological function of m^6^A in PCa we examined the mutational and expression status of proteins composing the MTC in a cohort of 492 PCa patients/samples from *The Cancer Genome Atlas* (TCGA) database. Inspection of the genomic regions encoding each one of the MTC subunits revealed that VIRMA is the most commonly altered MTC protein in PCa (8% of the samples), whereas the other MTC subunits disclose lower frequencies of alterations (1% for METTL3, 3% for METTL14, and 1.8% for WTAP) (Figure 1A). In most cases, VIRMA was amplified, whereas strong downregulation was most common for the other subunits. Next, we examined MTC subunit expression levels in TCGA and GTEx cohorts [16]. The GTEx cohort was added to increase the number of normal samples, which are underrepresented in the TCGA cohort. The results demonstrated high mRNA expression levels of VIRMA in at least 25% of the tumor samples compared with normal controls (Figure 1B), indicating that genomic amplification is not the only mechanism determining VIRMA overexpression. To investigate for possible associations between VIRMA overexpression and PCa progression, survival analysis was also performed using the available data. Remarkably, although differences in VIRMA mRNA expression levels were not statistically significant (one-way ANOVA test), patients with high VIRMA expression endured significantly shorter disease-free survival (Hazard Ratio 2.7, *p* = 0.0043) (Figure 1C), suggesting clinical significance of VIRMA overexpression in PCa.

### 2.2. m^6^A RNA Methylation and VIRMA are Upregulated in Hormone-Insensitive PCa Cell Lines

**Figure 2:**
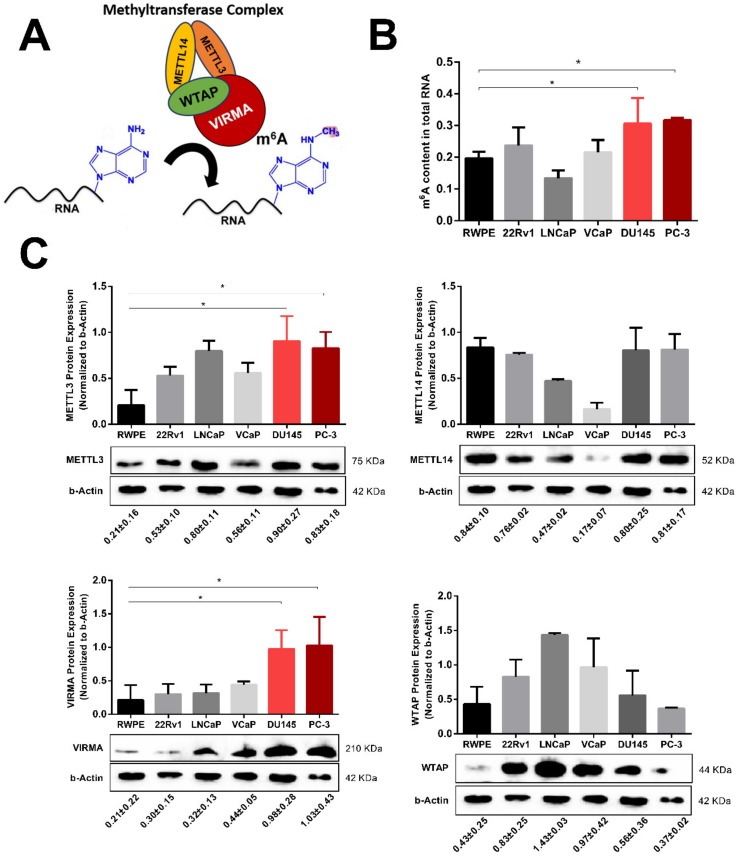
m^6^A RNA methylation is increased in androgen-independent prostate cancer cells. (**A**) Schematic illustration representing the methyltransferase complex (MTC) subunits that establish the m^6^A methylation mark; (**B**) m^6^A modification levels of total RNA assessed by ELISA in normal prostate basal epithelium cells (RWPE) and 5 different PCa cell lines (22RV1, LNCaP, VCaP, Du145, PC-3); (**C**) Western blot analysis showing the protein levels of each of the MTC subunits in the same cells. Shown is the relative protein abundance (normalized to β-actin). Data are shown as means ± SD and are representative of at least three independent experiments. * *p* < 0.05, Mann–Whitney *U* test.

To investigate the biological role of m^6^A RNA methylation in PCa, we quantified global m^6^A level in five human PCa cell lines (two androgen-dependent cell lines, LNCaP and VCaP) and three cell lines with androgen-independent growth, 22Rv1, DU145 and PC-3) and one benign prostate basal epithelium cell line (RWPE). Colorimetric m^6^A quantification assay showed higher m^6^A levels in androgen-independent versus androgen-dependent cells. Furthermore, androgen-independent DU145 and PC-3 PCa cells showed significantly increased m^6^A compared to RWPE cells (*p* = 0.0310 and *p* = 0.0160, respectively) (Figure 2B). Owing to the pivotal role of MTC in m^6^A modification (Figure 2A), we expected that increased m^6^A levels might be caused by alterations of m^6^A “writer” enzymes. To explore which one of the “writers” would be responsible for increased m^6^A levels in androgen-independent PCa cells, we assessed the protein levels of each enzyme using Western blot. The expression of VIRMA and associated levels of global m^6^A methylation in DU145 and PC-3 cancer cells were significantly higher compared to normal prostate cells (*p* = 0.0434 and *p* = 0.0298, respectively) (Figure 2C). The catalytic MTC subunit, METTL3 followed a similar pattern although to a lesser extent, suggesting that VIRMA might be the “writer” responsible for increased m^6^A methylation in advanced PCa.

### 2.3. VIRMA Knockdown in PC-3 Causes m^6^A Reduction and Attenuate Cells Malignancy

**Figure 3:**
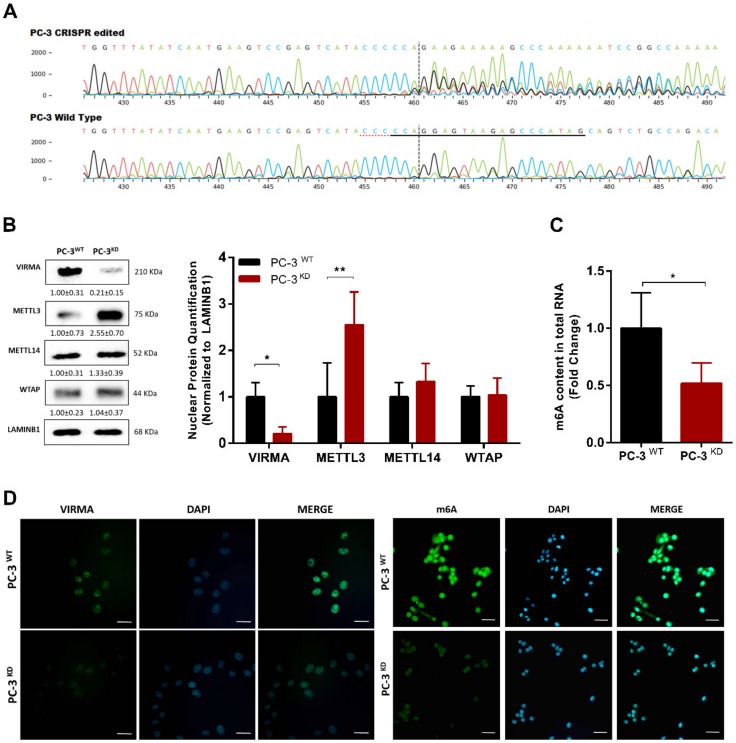
VIRMA knockdown in PC-3 cells caused global m^6^A reduction. (**A**) Sanger sequencing of PC-3 Wild Type (control) and CRISPR edited sequences in the regions around the guide sequence (the horizontal black underlined region represents the RNA guide sequence, the horizontal red underline is the PAM site and the vertical black dotted line represents the cut site); (**B**) Western blot quantification of the total amount of METTL3, METTL14, WTAP, and VIRMA in the nuclear protein fraction after VIRMA knockdown. Relative protein abundance was determined by normalization with LAMINB1. Bars represent mean ± SD based on 3 independent experiments, * *p* < 0.05, Mann–Whitney *U* test. (**C**) Global m^6^A modification levels in total RNA after VIRMA silencing in PC-3 cells, * *p* < 0.05 and ** *p* < 0.001, Mann–Whitney *U* test; (**D**) VIRMA expression and m^6^A levels assessed by confocal immunofluorescent assay (scale bar = 20 µm).

To investigate whether VIRMA overexpression may directly cause increased m^6^A methylation levels in PCa and whether it associates with cellular features of aggressive phenotype, we used a CRISPR/Cas9 approach. CRISPR knockdown PC-3 cell line (PC-3^KD^) (Figure 3A) was purchased from Synthego Corporation. Assessment of protein levels of each of the MTC units demonstrated that VIRMA nuclear protein levels were significantly reduced to 21% in PC-3^KD^ compared to wild type PC3 cells (PC-3^WT^) (Mann–Whitney *U* test, *p* < 0.05). In contrast, levels of METTL3 catalytic unit increased significantly—more than 2.5-fold (Mann–Whitney *U* test, *p* < 0.01)—in PC-3^KD^ (Figure 3B). 

Importantly, VIRMA depletion in PC-3 cells significantly decreased RNA m^6^A levels assessed by immunofluorescence (Figure 3D) and global RNA methylation quantification estimated by colorimetric assay (*p* = 0.0317) (Figure 3C). These findings strongly suggest that VIRMA knockdown triggers a compensatory feedback loop to enhance the expression of the catalytic METTL3 subunit. Nonetheless, compensatory overexpression of METTL3 is insufficient to preserve MTC functionality in the absence of VIRMA.

**Figure 4:**
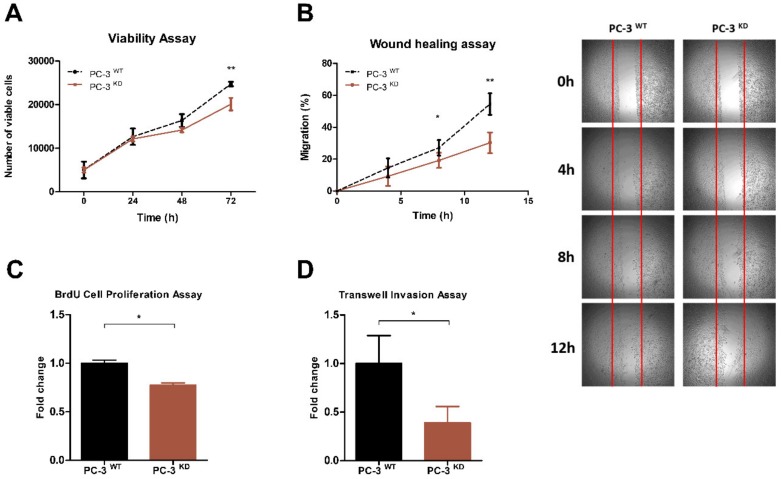
VIRMA expression promotes PC-3 cells growth and progression in vitro. (**A**) MTT assay for the number of viable PC-3^WT^ and PC-3^KD^ cells at 24 h, 48 h, and 72 h after seeding. (**B**) Quantitation of Wound-healing assay for PC-3^WT^ and PC-3^KD^. Points and connecting line on the left panel represent the migration index of wound-healing assay over the course of 12 h. The distance migrated by PC-3^KD^ cells is represented as relative to that migrated by PC-3^WT^ cells in the same time period. Representative photos are shown in the panel on the right. (**C**) Proliferation of PC-3^WT^ and PC-3^KD^ cells assessed by BrdU assay at 24 h. (**D**) Invasion of PC-3^WT^ and PC-3^KD^ cells assessed by Matrigel transwell assay at 24 h. Column bars in (C) and (D) represent the average number of cells from 3 independent experiments. Error bars represent ± SD. ** *p* < 0.001 * *p* < 0.05, Mann–Whitney *U* test.

Next, we investigated whether the reduction of global m^6^A methylation levels affects PCa cells growth characteristics and malignant phenotype (Figure 4). VIRMA knockdown significantly decreased viability of PC3^KD^ cells 72 h after transfection (20%, *p* < 0.01). Migration of PC3^KD^ cells in wound healing assay was also significantly inhibited at 8h (*p* < 0.05) and even more evident at 12 h (*p* < 0.01). It should be noted, however, that this effect might also be due to reduction of PC-3^KD^ cells proliferation (25%, *p* < 0.05) after VIRMA knockdown. Invasion capacity is also significantly decreased in PC-3^KD^ cells comparatively to wild-type (60%, *p* < 0.05). 

### 2.4. m^6^A Downregulation Suppresses Oncogenic Long Non-Coding RNAs Expression

It has been shown that m^6^A residues within the transcript body of lncRNAs may influence their biological function [17]. As lncRNAs are important players in PCa progression [18] we sought to examine the effects of m^6^A depletion on lncRNA expression. Using RT^2^ Profiler PCR array we tested for altered expression levels of a panel 84 cancer-associated lncRNAs, between PC-3^WT^ and PC-3^KD^ cells.

**Figure 5:**
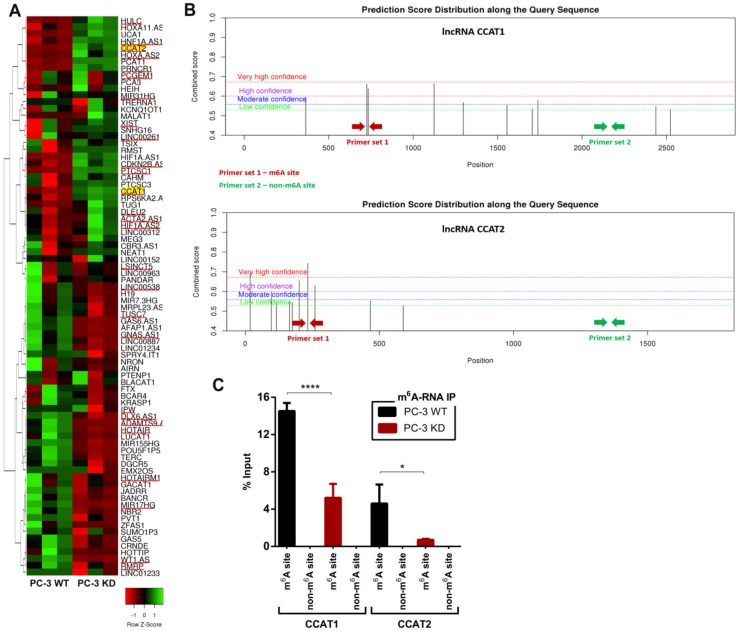
VIRMA expression endorses long non-coding RNAs (lncRNAs) expression in m^6^A-dependent way. (**A**) Heat map and clustering analysis of lncRNAs expression in PC-3^WT^ and PC-3^KD^ cells, 3 biological replicates per condition. The 27 lncRNAs differentially expressed are underline red and the selected ones are further highlighted in yellow; (**B**) Potential m^6^A binding sites at lncRNAs CCAT1 and CCAT2 predicted with high and very high confidence (red and purple lines) by SRAMP (http://www.cuilab.cn/sramp/) [19]. Red and green arrows indicate the regions corresponding to PCR amplicons used for lncRNA quantification in immunoprecipitation experiments; (**C**) Abundance CCAT1 and CCAT2 transcripts in m^6^A-RNA IP pools from PC-3^WT^ and PC-3^KD^ cells. After purification precipitated RNA was used as a template for reverse transcription and real-time PCR with primers as indicated in 5b. Data is represented as percentage of input; **** *p* < 0.0001 and * *p* < 0.05, Mann–Whitney *U* test.

We identified 27 differentially expressed lncRNAs in PC-3^KD^ compared to PC-3^WT^ (Figure 5A and Table S1). From those, 17 lncRNAs were significantly downregulated in PC-3^KD^ cells (*p* < 0.00001, Student’s *t*-test). Within this group, lncRNAs CCAT1 and CCAT2 displayed the strongest downregulation upon VIRMA depletion (fold change –3.7 and –8.5, respectively) and were selected for further validation. 

Next, we identified putative m^6^A sites based on the presence of the DRACH consensus motifs in the sequence of CCAT1 and CCAT2 transcripts (Figure 5B). Although 10 predicted m^6^A sites were found for each of the transcripts, in CCAT1 the distribution was relatively uniform along the transcript body, whereas in CCAT2 they were mainly located at the 5′-terminal regions of the transcript. Specific primers targeting the regions with highest m^6^A methylation probability, as well as control non-methylated regions (Figure 5B), were designed to assess the number of CCAT1/2 transcripts immunoprecipitated from PC-3^WT^ and PC-3^KD^ cells. As expected, VIRMA knockdown decreased the number of CCAT1 and CCAT2 transcripts with detectable m^6^A methylation in the predicted targeted regions (*p* < 0.0001 and *p* < 0.05) (Figure 5C).

**Figure 6:**
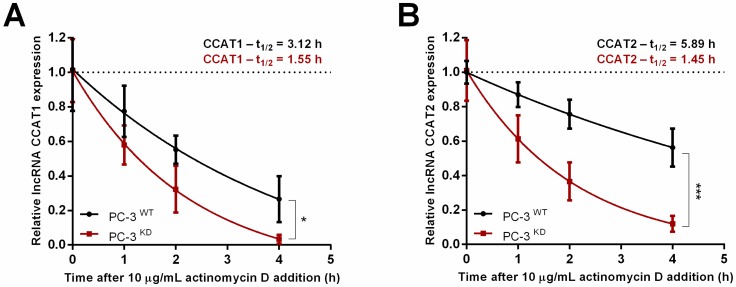
m^6^A stabilizes CCAT1 and CCAT2 lncRNAs. VIRMA knockdown decreases the stability of lncRNA CCAT1 (**A**) and CCAT2 (**B**) in PC-3 cells upon treatment with Actinomycin D, * *p* < 0.05 and *** *p* < 0.0001, Sum of squares *F* test.

To dissect the mechanism by which m^6^A controls CCAT1/2 expression, we measured the stability of both lncRNAs. PC-3^WT^ and PC-3^KD^ cells were treated with 10 µg/mL actinomycin D, an inhibitor of RNA polymerase elongation. Over the course of 4 h, we observed a faster decay rate for both CCAT1 and CCAT2 lncRNAs in the absence of m^6^A methylation. To calculate the half-life of the tested lncRNAs we fitted the data into a non-linear regression model. CCAT1 half-life in PC-3^KD^ cells was moderately reduced by 50% (*p* < 0.05) after 4 h of treatment, whereas half-life reduction was more expressive for CCAT2 (*p* < 0.0001), i.e., 76% after 4 h (Figure 6). Notably, differences between PC-3^WT^ and PC-3^KD^ decay rates were more pronounced for CCAT2 transcript, suggesting that the distribution of m^6^A is relevant for transcript stability.

### 2.5. Higher VIRMA and lncRNAs Expression Predicts Poor Prognosis in PCa Patients

**Figure 7:**
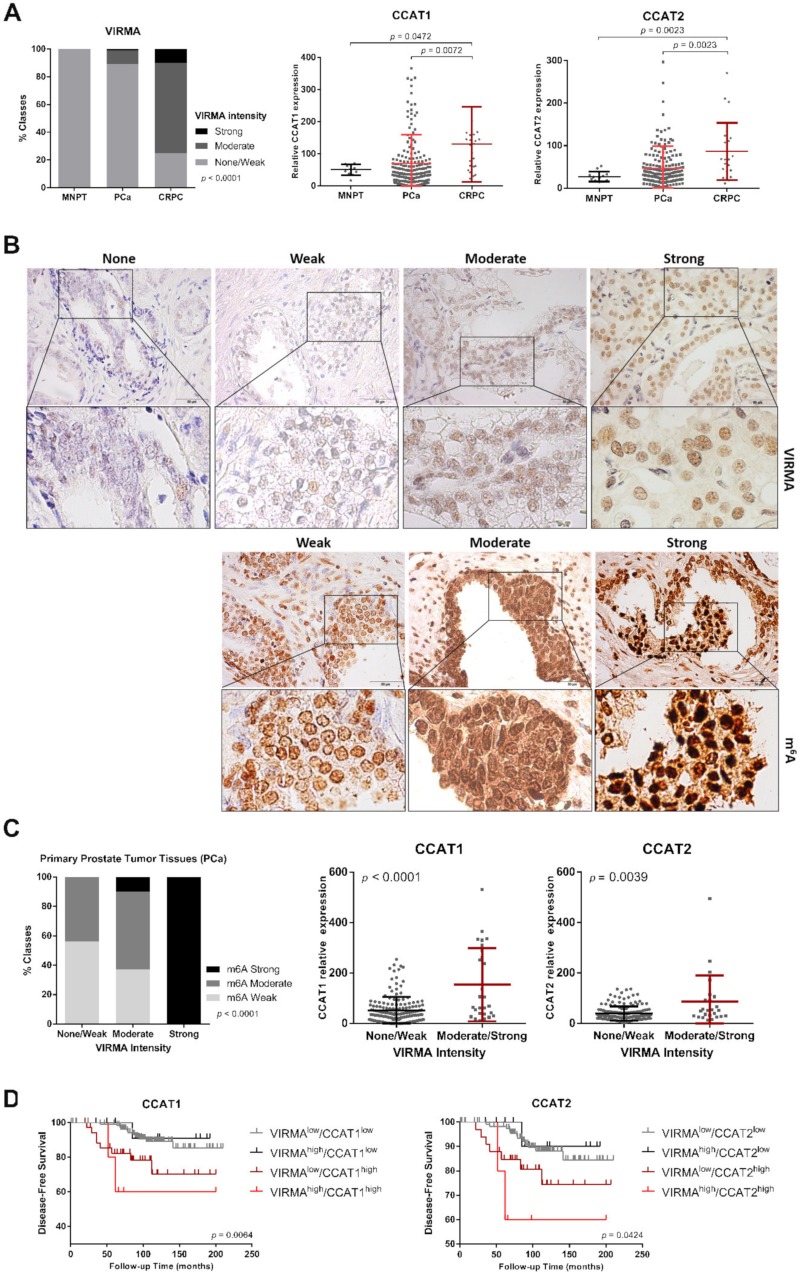
VIRMA, CCAT1, and CCAT2 are overexpressed in PCa tissues and associate with poor prognosis. (**A**) On the left, the contingency graph displaying the immunostaining intensity of VIRMA (chi-square test) and, on the right, the scatter plot representing the relative lncRNA expression (Mann–Whitney *U* test) in morphologically normal prostate tissue (MNPT), hormone-naïve PCa (PCa) and castration-resistant PCa (CRPC); (**B**) Representative images of VIRMA and m^6^A immunostaining in the IPO-Porto patient cohort; (**C**) m^6^A immunostaining intensity and relative lncRNA expression in hormone-naïve PCa tissues stratified according to VIRMA immunostaining intensity; (**D**) Kaplan–Meier survival curves of VIRMA and CCAT1/2, log-rank test.

To evaluate whether our findings might hold clinical significance, an independent patient cohort of age matched prostate tissues comprising 198 hormone-naïve PCa, 24 CRPC and 13 morphologically normal prostates (MNPT), all collected at Portuguese Oncology Institute of Porto (IPO Porto), was examined. Immunohistochemistry was performed to evaluate m^6^A levels and VIRMA protein expression; lncRNA expression was measured using qPCR. Interestingly, VIRMA and lncRNAs CCAT1/2 disclosed higher expression in PCa tissues and were particularly upregulated in CRPC cases compared to hormone-naïve PCa (Figure 7A). 

In hormone-naïve PCa, VIRMA expression and cellular m^6^A methylation levels were positively associated (chi-square test, *p* < 0.0001). Moreover, samples displaying moderate and strong VIRMA intensity had significantly higher CCAT1/2 lncRNA expression levels compared to samples with weak or absent VIRMA immunoexpression (Mann–Whitney *U* test, *p* < 0.0001 and *p* = 0.0039, respectively) (Figure 7B,C). 

Because lncRNA CCAT1 and CCAT2 are involved in regulation of proto-oncogene *MYC* [20,21], the link between VIRMA-dependent m^6^A-regulated lncRNAs and their ability to increase *MYC* expression was also tested in these patient samples. Remarkably, a moderate/weak positive correlation was observed between CCAT1/2 and *MYC* transcript levels (Spearman *r* = 0.3 *p* < 0.0001 for CCAT1; Spearman *r* = 0.4 *p* < 0.0001 for CCAT2). 

To evaluate the association with clinical parameters, we examined the expression of VIRMA and CCAT1/2 and compared it with standard clinicopathological data (Table S2) by univariate and multivariate analysis. Remarkably, patients with higher VIRMA and lncRNAs CCAT1/2 disclosed worse prognosis and shorter time to biochemical relapse (Figure 7D). Interestingly, higher expression of VIRMA and both lncRNAs (as a group variable) independently predicted shorter disease-free survival (HR = 9.083, CI95% 1.911–43.183, *p* = 0.006) (Table 1).

## 3. Discussion

PCa-related deaths are mostly due to advanced disease, refractory to currently used therapies; hence, uncovering the mechanisms involved in high-risk and castration-resistant PCa remains a challenge [22] and further research in this area is imperative. Emerging evidence demonstrates that dysregulation of the molecular machineries involved in RNA modification, and, specifically, of the complexes involved in m^6^A RNA methylation, may lead to cancer progression [23,24,25]. Nevertheless, findings regarding m^6^A function in tumor progression are still under debate as the involvement of individual protein factors appears to be tissue and context specific. For instance, m^6^A demethylase ALKBH5 was reported to induce gastric cancer metastasis by demethylating lncRNA NEAT1 [26], whereas an earlier study showed an important role for ALKBH5 in inhibiting pancreatic cancer progression [27]. Similarly, METTL14 seems to play an oncogenic function in acute myeloid leukemia [28], but in hepatocellular carcinoma, it behaves as metastasis suppressor by modulating m^6^A dependent microRNA processing [24]. Regarding m^6^A role in PCa, although still largely unexplored, overexpression of the m^6^A reader YTHDF2 has been associated with malignant progression [29]. 

Herein, we aimed to assess the role of m^6^A and MTC in PCa. TCGA dataset was examined, and VIRMA was identified as the most frequently altered gene of the complex involved in m^6^A establishment. VIRMA was often amplified, reflecting higher expression in cancer compared with normal tissues. Also, PCa patients with high VIRMA expression endured significantly shorter disease-free survival (DFS). No similar trend was observed for the other complex subunits (METTL3, METTL14 and WTAP). Although all four proteins participate together as MTC subunits, each of them can function independently in other cellular processes [5,30]. Thus, further characterization in a set of PCa cell lines and in an independent patient cohort was carried out. Our findings confirmed VIRMA overexpression and demonstrated increased m^6^A RNA methylation in androgen-independent PCa cells and CRPC samples, compared to normal cells/tissues. Remarkably, the higher percentage of amplification, and consequently expression, disclosed for VIRMA compared to the other core subunits might be explained by VIRMA genomic location at 8q chromosome, which is commonly altered in locally advanced and metastatic prostate cancers [31,32].

VIRMA is important for the proper establishment of the cellular m^6^A profile [33] and, indeed, its knockdown in the metastatic androgen-independent PC-3 cells led to significant m^6^A reduction. Recently, similar results were reported for METTL3 silencing in PCa cell lines [34]. Proteomic analyses established that VIRMA mediates the activity of MTC [35] whereas experimental evidence in Hela cells demonstrated functional protein-protein interaction between VIRMA and the other MTC core subunits METTL3, METTL14, and WTAP [7]. Based on these findings, we hypothesized that the same interaction might also occur in PCa cells and that once VIRMA is depleted, access of methyltransferases to specific RNA substrate is significantly reduced. However, additional studies are needed to further confirm our theory in PCa cell lines. 

At the cellular level, VIRMA knockdown significantly inhibited the viability and proliferation of PC-3 cells and abated the malignant phenotype by reducing migration and invasion properties. In other tumor models, downregulation of m^6^A methylation is also associated with attenuation of malignant features through impairment of epithelial mesenchymal transition of cancer cells and translation of Snail [36]. VIRMA is also reported as regulator of CDK1 mRNA expression, an important cell cycle factor, in an m^6^A-independent manner [37]. Future research should clarify whether these downstream effects also occur in PCa. Globally, these findings indicate that VIRMA depletion mitigates cancer progression by decreasing m^6^A RNA methylation levels in PCa, similarly to other cancer types, such as gastric, breast and hepatocellular carcinomas [23,37,38].

The full extent of mechanisms by which m^6^A marks can modulate the function of mRNA and lncRNA remains to be elucidated. M^6^A residues can produce local change in the RNA scaffold that may alter RNA folding and affect the interaction with proteins and other RNAs [39,40]. Although most of the m^6^A studies focused on its role in mRNA function, recent reports showed that m^6^A may also regulate ncRNAs functions [17]. Similarly to mRNAs, lncRNAs are also dynamically m^6^A methylated and the modification levels depend on the cell line, tissue type and growth conditions [41,42]. In this study, we focused on potentially m^6^A-regulated lncRNAs with impact on PCa. Our results demonstrate that in PC-3 cells the cancer-associated lncRNAs CCAT1 and CCAT2 are specifically m^6^A methylated. Additionally, the reduction of m^6^A modification at lncRNAs’ specific sites negatively affects their stability, decreasing expression, and ultimately contributing to a less aggressive PCa phenotype. Remarkably, other studies also demonstrated that CCAT1 or CCAT2 knockdown through siRNAs inhibit cell growth, migration, and invasion in PC-3 and DU145 cells [21,43], indicating that CCAT1/2 silencing mimics VIRMA knockdown in metastatic androgen-independent PCa cells. 

Interestingly, CCAT2 displayed a faster reduction rate than CCAT1 after Actinomycin D treatment in m^6^A depleted cells. Transcripts from distinct genes are degraded at substantially different rates and RNA modifications may alter these rates by influencing mRNA’s accumulation and steady-state abundance [44]. Indeed, transcript stability is highly dependent on m^6^A readers and the RNA-binding proteins that interact with them. Insulin-like growth factor 2 mRNA-binding proteins 1–3 (IGF2BP1-3), were found to stabilize target RNA in an m^6^A-dependent manner [45], and density and sequence contexts of m^6^A sites are also likely to be relevant [5]. Future CLASH experiments [46] may provide a more complete and detailed answer to this question. The preferential grouping of m^6^A sites in the first part of CCAT2 transcript body may contribute to a m^6^A-switch model, in which m^6^A regulates protein binding through its influence on RNA structure, as previously described for MALAT1 [47]. Thus, CCAT2 might be more prone to benefit from interaction with m^6^A reader proteins which could stabilize its structure and protect the transcript from RNA cleavage. 

**Figure 8:**
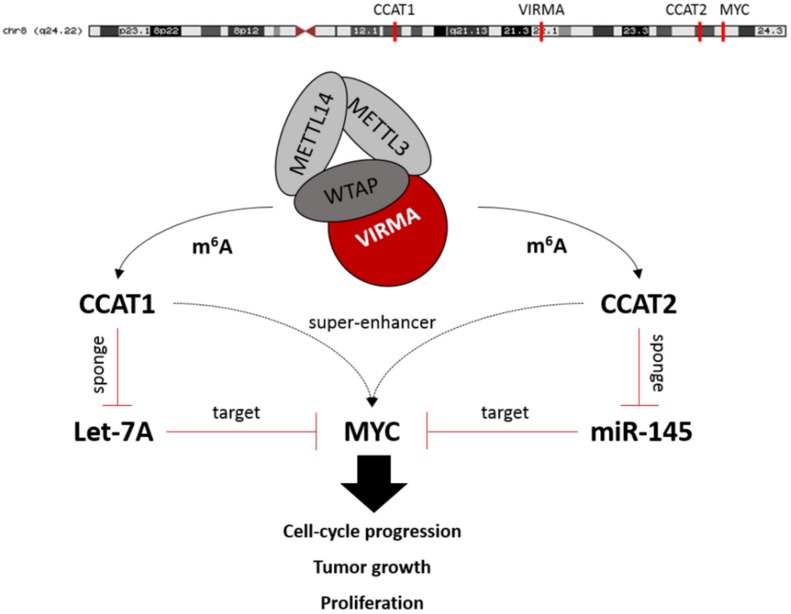
Proposed mechanism of the interplay between the putative VIRMA-dependent m^6^A-regulated CCAT1/2 lncRNAs and *MYC* proto-oncogene.

CCAT1 and CCAT2 were discovered as colorectal cancer associated transcripts but have also been shown to be involved in prostate tumorigenesis, among others [43,48]. In fact, in our set of primary tissue samples we found a direct correlation between CCAT1/2 and *MYC* transcript levels, which plays an oncogenic role in PCa [49]. In view of these results and currently available data, we postulate that stabilization of CCAT1/2 by m^6^A has an amplifying effect on *MYC* expression levels in cancer cells via 2 separate mechanisms: (i) Directly, through both lncRNAs acting as super-enhancers that positively regulate *MYC* mRNA [21]; (ii) Indirectly, by means of CCAT1/2 acting as microRNA sponges for *MYC*-targeting microRNAs let-7A and miR-145, respectively [50,51,52,53] (Figure 8). Accordingly, a moderate but highly significant correlation between CCTA1/2 and *MYC* transcript expression was depicted in our clinical samples set. It should be emphasized that this correlation may also be caused by the co-localization of all 4 genes (*VIRMA*, *CCAT1*, *CCAT2*, *MYC*) at 8q chromosome, which is commonly amplified in PCa, a feature that deserves future investigation.

Lastly, we confirmed that VIRMA expression is significantly associated with CCAT1/2 cellular m^6^A levels and expression in primary PCa tissues. Subsequent examination of CCAT1/2 expression in IPO-Porto tissue set confirmed that high lncRNA expression levels are a negative prognostic factor, impacting on DFS, in PCa patients and that the addition of VIRMA expression to the model significantly improves its predictive power. Our study is the first to report VIRMA overexpression, at protein level in PCa tissues, particularly in those from patients with castration-resistant disease. 

Summarizing, our results suggest that VIRMA is involved in molecular mechanisms that may contribute to the development of mCRPC through m^6^A deregulation. We further showed that depletion of this methylation mark from cancer-associated lncRNAs specifically affects their expression. Although we have emphasized the importance of VIRMA and lncRNAs CCAT1 and CCAT2 methylation in high-risk PCa using molecular biology strategies, limitations must also be acknowledged. Indeed, in vivo experiments would be beneficial for the validation of these results in a more complex system. Furthermore, considering the observed associations between VIRMA, selected lncRNAs and clinical determinants of tumor behavior, assessment of expression levels in liquid biopsies, such as urine and plasma, should be performed in the future to investigate their potential use as prognostic markers, aiding clinical decision making and management of PCa patients.

## 4. Materials and Methods

### 4.1. Bioinformatics Analysis

In silico analysis of the integrity of the genomic regions encoding each one of the MTC subunits was performed on the PCa cohort (492 patients/samples) publicly available at *The Cancer Genome Atlas* (TCGA) database. The online resource *cBioPortal for Cancer Genomics* (https://www.cbioportal.org/; last access on 14/02/2020) [54] was used with the user-defined entry gene set “METTL3, METTL14, WTAP, VIRMA”.

RNA sequencing expression data based on TCGA and also in *Genotype-Tissue Expression* (GTEx) projects were analyzed with an interactive web server, the *Gene Expression Profiling Interactive Analysis* (GEPIA) server using default parameters (http://gepia.cancer-pku.cn/; last access on 14/02/2020) [16]. A total of 492 prostate adenocarcinoma (PRAD) and 152 normal adjacent tissue samples were analyzed for the expression of key genes involved in m^6^A establishment. For the generation of dot plots and for ANOVA, genes were considered differentially expressed when expression levels were changed at least two fold (log2 fold-change threshold value equal to or bigger than 1) and had an Benjamini and Hochberg false discovery rate adjusted *q*-value smaller then 0.01. For survival plots, samples were binned based on quartiles. Samples with expression levels of METTL3, METTL14, WTAP, and VIRMA, higher than 75^th^ percentile were included in the high-expression cohort while samples with expression levels of METTL3, METTL14, WTAP, and VIRMA, lower than 25^th^ percentile were included the low-expression cohort. All other samples were excluded of further analysis. Mantel–Cox test was used for hypothesis test.

### 4.2. Cell Lines and Cell Culture

Human PCa cell lines available in our lab (22Rv1, LNCaP, VCaP, DU145, and PC-3, all from ATCC) and a non-malignant prostate epithelial cell line (RWPE, kindly provided by Prof. Margarida Fardilha, University of Aveiro, Portugal) were used in this study. Genetically modified PC-3 VIRMA CRISPR knockdown (PC-3^KD^) was generated by Synthego Corporation with the following guide RNA sequence: 5′ CUAUGGGCUCUUACUCCUGG 3′. Cell culture were performed accordingly [55].

### 4.3. RNA Methylation Quantification

RNA was extracted with Trizol reagent (Invitrogen, Carlsbad, CA, USA) according to manufacturer’s instructions. The m^6^A RNA methylation quantification kit (ab185912; Abcam, Cambridge, United Kingdom) was used to measure the m^6^A content of total RNA following the manufacturer’s instructions. A standard curve was built from synthetic RNAs provided in the kit to infer the m^6^A levels.

### 4.4. Protein Extraction and Western Blot Analysis

Total nuclear protein was extracted using Nuclear Extract Kit (Active Motif, Carlsbad, CA, USA). Total cellular protein was extracted using the radioimmune precipitation assay buffer (RIPA) (Santa Cruz Biotechnology Inc, Dallas, TX, USA) with 10% Protease Inhibitor Cocktail (Merck Millipore, Burlington, MA, USA). Western blot analysis was performed as described in [55]. Primary antibodies used and respective dilutions and incubation times are depicted in Table S3. All blot images from direct Western Blot were captured by Bio-Rad ChemiDoc-Western Blot Digital Imaging System and the intensity of each band as well as respective ratio were quantified using Quantity One software (Bio-Rad Laboratories, Hercules, CA, USA), by comparing the protein band intensity with the loading control (β-Actin). 

### 4.5. Immunofluorescence Analysis

Cells were fixed with 4% paraformaldehyde, permeabilized with 0.25% Triton-X and blocked with 5% bovine serum albumin at room temperature. Fixed cells were incubated firstly with primary antibody (Table S3) and then with secondary antibody anti-rabbit immunoglobulin G (Alexa FluorTM 488 goat, A11008, Invitrogen, Carlsbad, CA, USA). DNA staining was performed with 4′,6-diamidino-2-phenylindole (Boster Biological Technology, Pleasanton, CA, USA). Pictures were taken in a fluorescence microscope Olympus IX51 (400× magnification) with digital camera Olympus XM10, using CellSens software.

### 4.6. Phenotypic Assays

To evaluate the impact of VIRMA knockdown in PC-3, 3-(4,5-dimethylthiazol-2-yl)-2,5-diphenyltetrazolium (MTT; Sigma-Aldrich, St. Louis, MO, USA) assay was performed. To determine differences in cell proliferation the Cell Proliferation ELISA BrdU assay (Roche Applied Sciences, Basel, Switzerland) was used. Invasion ability of cells was analyzed using BD Biocoat™ Matrigel Invasion Chambers (BD Biosciences, San Jose, CA, USA). The procedures followed the published report [23]. For wound-healing assay cells were seeded and grown to 100% confluence and then scratched with a sterile 200 μL pipette tip to create an artificial wound. At 0, 4, 8, and 12 h after wounding, phase-contrast images of the wound healing process were photographed with a 10x objective lens. Eight images per condition were analyzed to determine averaging position of the migrating cells at the wound edges.

### 4.7. RT² lncRNA PCR Array Human Cancer PathwayFinder

First-strand cDNA synthesis was done with Quantitect Reverse Transcription (Qiagen Hilden, Germany). Real-time PCR was then performed with the commercially available Human Cancer Pathway Finder RT^2^ Profiler PCR array (Qiagen, Hilden, Germany) according to the manufacturer’s instructions. *p*-value < 0.0001 (Student’s *t*-test) and a fold change >3.0 were considered to indicate significant dysregulation.

### 4.8. N6-Methyladenosine Immunoprecipitation

Immunoprecipitation of m^6^A was adapted from the EpiMark^®^ N6-Methyladenosine Enrichment Kit (New England Biolabs, Ipswich, MA, USA). RNA samples were fragmented by heating (95 °C 3 min) prior to immunoprecipitation. After immunoprecipitation, RNA was eluted subsequently purified using Purelink RNA Mini Kit (Ambion, Waltham, MA, USA), according to manufacture instructions. For quantification of RNA immunoprecipitation, both input samples and IP elutes were examined by qRT-PCR. Primer sequences are presented in Table S4. To analyze the results, the percentage input method was used [100 × 2^ (Adjusted input − Ct (IP)].

### 4.9. Actinomycin D Assay

PC-3^WT^ and PC-3^KD^ cells were grown in adequate medium prior addition of 10 µg/mL actinomycin D (Sigma-Aldrich, St. Louis, MO, USA), harvested at 4 different time points and used for RNA isolation. LncRNAs levels were determined using RT-qPCR with the same primers as in Table 1. The data were analyzed using the 2-ΔΔCt method and GUSβ mRNA was used as an endogenous control. Relative lncRNA levels were plotted against time after actinomycin D addition and fitted to a non-linear regression model in order to calculate CCAT1 and CCTA2 lncRNAs’ half-life.

### 4.10. Tissues and Ethical Statement

Tissues specimens from 198 patients harboring clinically localized prostate carcinoma (PCa), 24 CRPC and 13 morphologically normal prostate tissue (MNPT) samples were collected at Portuguese Oncology Institute of Porto (IPO-Porto). Histological slides from formalin-fixed paraffin-embedded tissue fragments were obtained from the surgical specimens and assessed for Gleason score and TNM stage. Relevant clinical data was collected from clinical charts. This study was approved by the institutional review board (Comissão de Ética para a Saúde-(CES-IPOFG-EPE 205/2013)) of IPO Porto.

### 4.11. Immunohistochemistry

Antigenic recovery was performed with EDTA buffer in water bath and endogenous peroxidase activity was blocked by 0.6% hydrogen peroxide. Nonspecific reactions were blocked with normal horse serum (dilution 1:50). Slides were incubated with the primary antibodies (Table S3) and with Novolink™ Polymer Detection System (Leica Biosystems, Wetzlar, Germany). Diaminobenzidine was used as chromogen and hematoxylin as counterstain. Normal testicle and normal brain tissue were used as positive controls for VIRMA and m^6^A, respectively. Immunoexpression intensity was estimated by an expert uropathologist, considering staining as “weak”, “moderate”, or “strong”.

### 4.12. Validation of Selected lncRNAs in Prostate Tissues

RNA was reverse transcribed and amplified using TransPlex Whole Transcriptome Amplification Kit (Sigma–Aldrich, St. Louis, MO, USA) with subsequent purification using QIAquick PCR Purification Kit (Qiagen, Hilden, Germany), according to manufacturer’s instructions. CCAT1 and CCAT2 levels were evaluated using Xpert Fast Sybr Master Mix (GRiSP Research Solutions, Porto, Portugal) and specific primers (Table S4). To determine the relative expression levels in each sample, the values of the target gene were normalized using an internal reference genes (GUSβ). 

### 4.13. Statistical Analysis

Data was tabulated using Microsoft Excel 2016 and analyzed using IBM SPSS Statistics version 24 (IBM-SPSS Inc., Chicago, IL, USA) and GraphPad Prism 7 (GraphPad Software Inc., La Jolla, CA, USA). Differences in relative expression among groups were assessed by the non-parametric Mann–Whitney *U* test. Associations between categorical variables were determined by means of a Chi-square test. Prognostic significance was assessed by the Kaplan–Meier curves with log-rank test. Cox regression model analysis was used to compare performance between PSA levels, pathological stage and VIRMA/lncRNAs expression as prognostic variables. Disease-free survival (DFS) was calculated from the date of the radical prostatectomy (primary treatment) to the date of biochemical relapse (2 PSA tests ≥ 0.2 ng/mL after prostatectomy), date of last follow-up, or death if relapse-free. For the purposes of survival analyses, all cases were coded based on the expression levels of each lncRNA or VIRMA expression using the 75^th^ percentile value as empirical threshold.

## 5. Conclusions

The methyltransferase associated protein VIRMA is the most commonly affected m^6^A writer in PCa cell line models and clinical samples. VIRMA is essential for the maintenance of m^6^A RNA methylation in PCa. Depletion of VIRMA in a mCRPC cell line model reduced global m^6^A levels leading to attenuation of phenotypic aggressive features. The removal of specific m^6^A marks from the investigated lncRNAs, CCAT1 and CCAT2, led to reduced transcript stability and abundance. 

These results provide new insights on the role of RNA methylation associated with prostate cancer aggressiveness and may assist in the development of novel prognostic markers for high-risk PCa and CRPC. The functional importance of lncRNA methylation in PCa is yet to be elucidated in full depth.

## Figures and Tables

**Table 1:** Multivariate Cox Regression analysis.

Disease-Free Survival (DFS)	Variable	HR	95% CI for HR	*p*-Value
**PSA levels**	<10 ng/mL	1 (referent)		0.086
	≥10 ng/mL	2.607	1.116–6.089	**0.027**
**Pathological stage (pT)**	pT2	1 (referent)		**0.028**
	pT3a	2.774	0.983–7.827	0.052
	pT3b	3.902	1.264–12.045	**0.018**
**VIRMA/CCAT1/CCAT2 expression**	VIRMA^low^/lncRNA^low^	1 (referent)		**0.033**
	VIRMA^low^/lncRNA^high^	2.475	0.978–6.259	**0.041**
	VIRMA^high^/lncRNA^high^	9.083	1.911–43.183	**0.006**

HR – Hazard ratio; CI – Confidence interval; Statistically significant *p*-values (<0.05) are highlighted in bold.

## Supplementary Materials

The following are available online at https://www.mdpi.com/2072-6694/12/4/771/s1; Table S1: RT² lncRNA PCR Array Human Cancer Pathway Finder data, Table S2: Clinical and pathological data of the fresh-frozen tissues included in this study, Table S3: Primary antibodies used in Western blot, immunofluorescence and immunohistochemistry, Table S4: Specific primer sequences. Full western blot images are provided in Supplementary information file.
