# Supplementary figures and images for: VIRMA-Dependent N6-Methyladenosine Modifications Regulate the Expression of Long Non-Coding RNAs CCAT1 and CCAT2 in Prostate Cancer
Source: Cancers (Basel). 2020 Mar 25;12(4):771. doi: 10.3390/cancers12040771 (PMC7226055; doi:10.3390/cancers12040771)

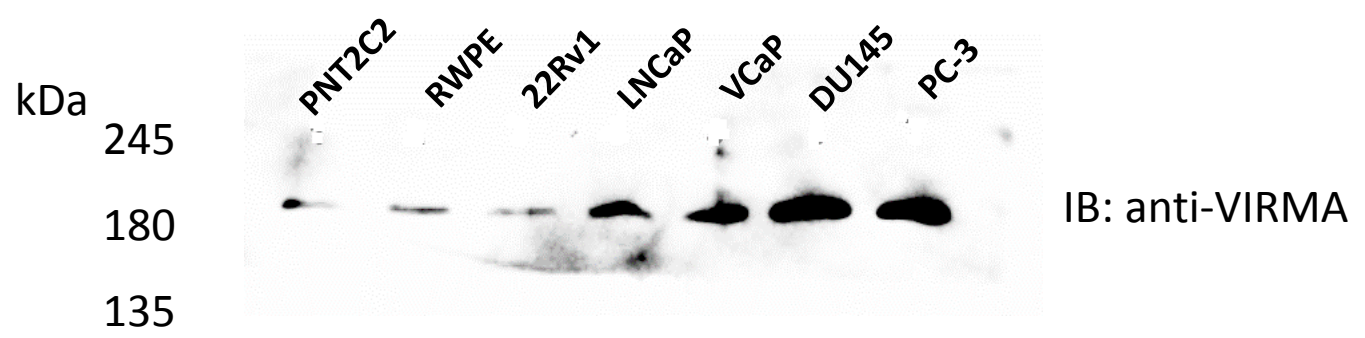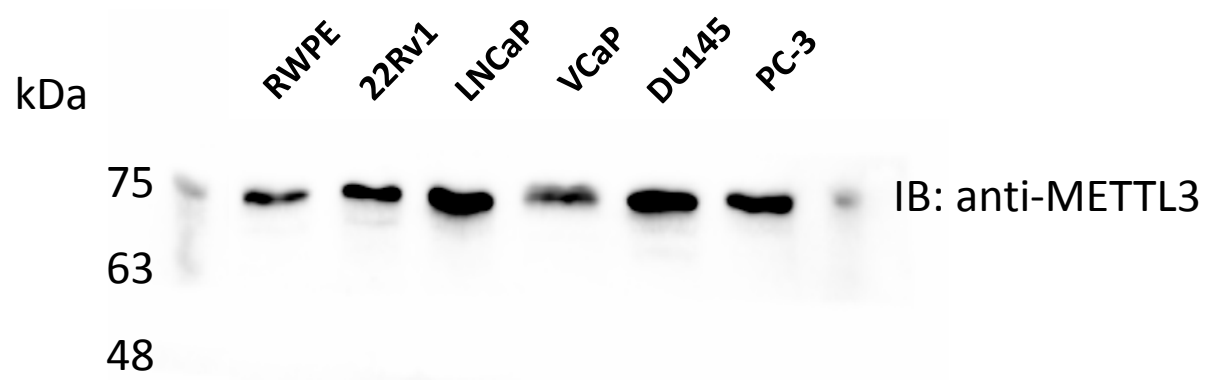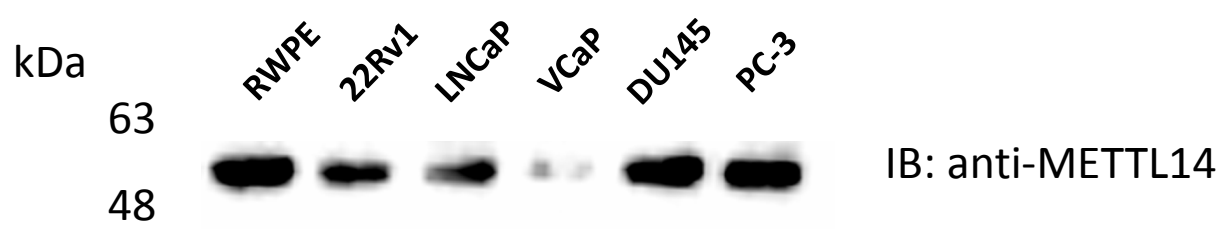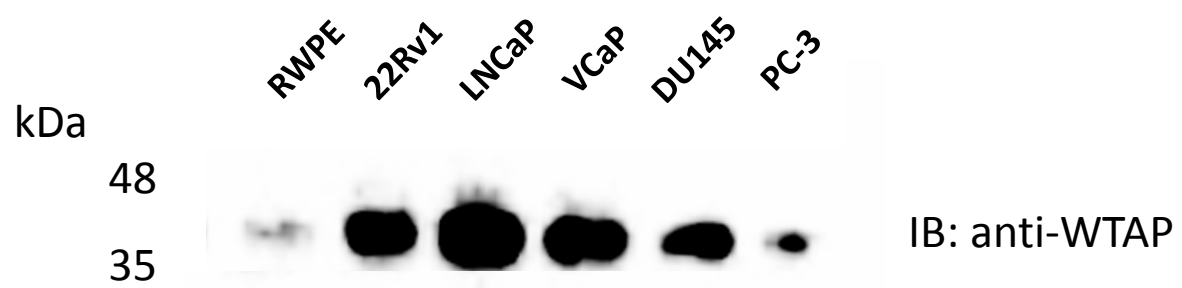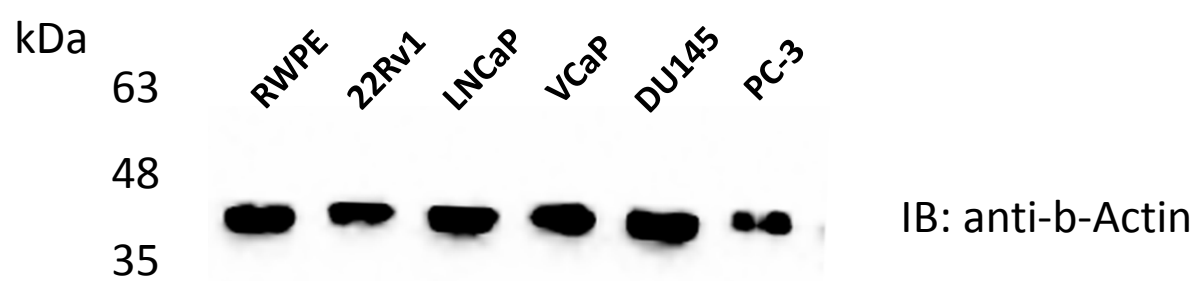

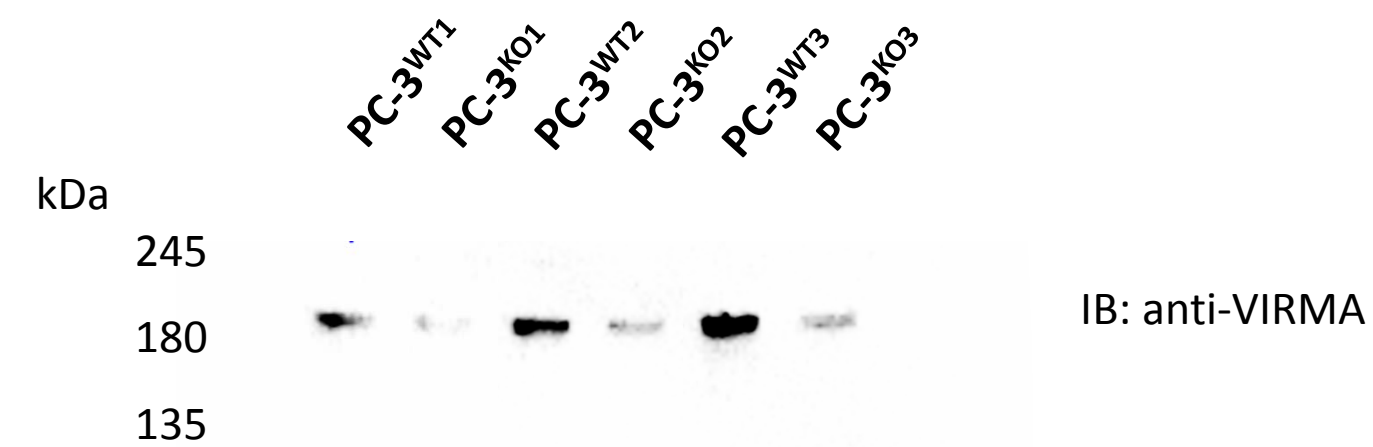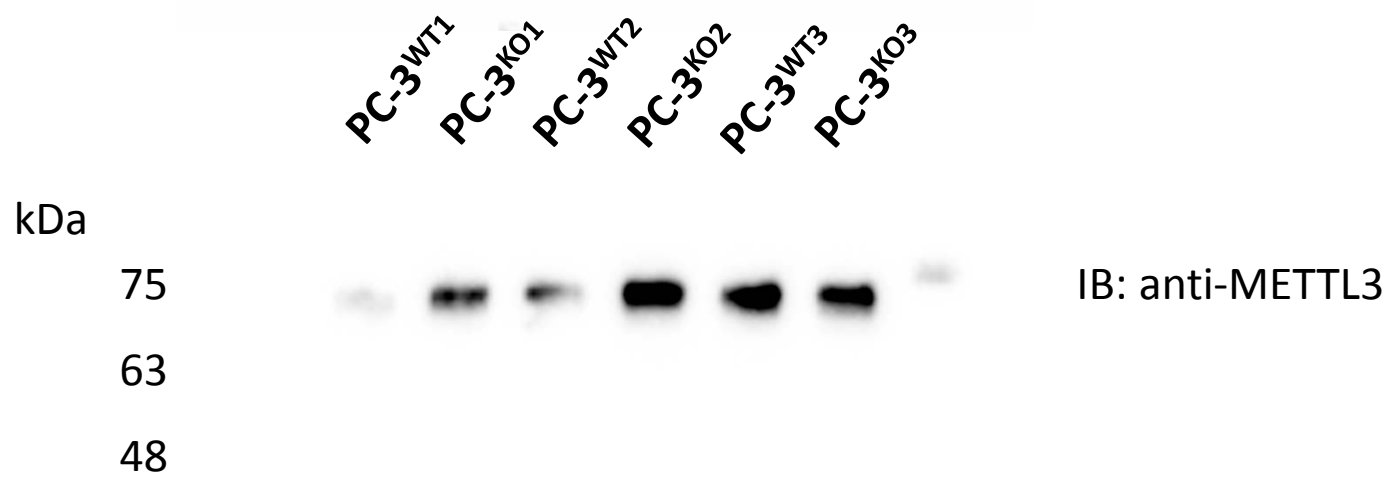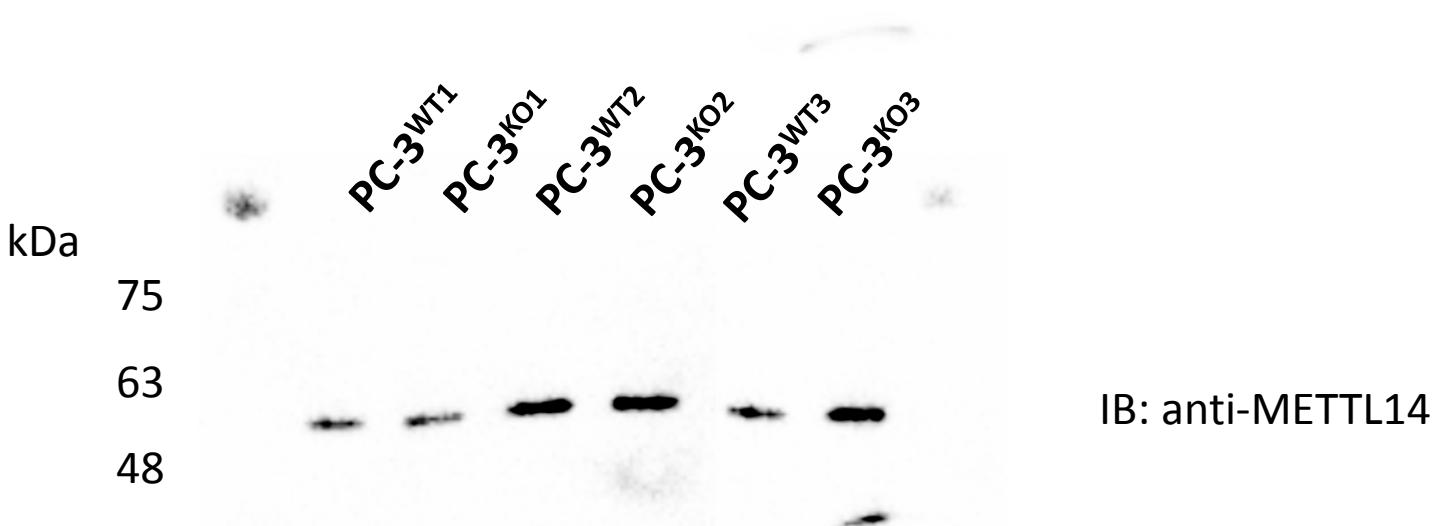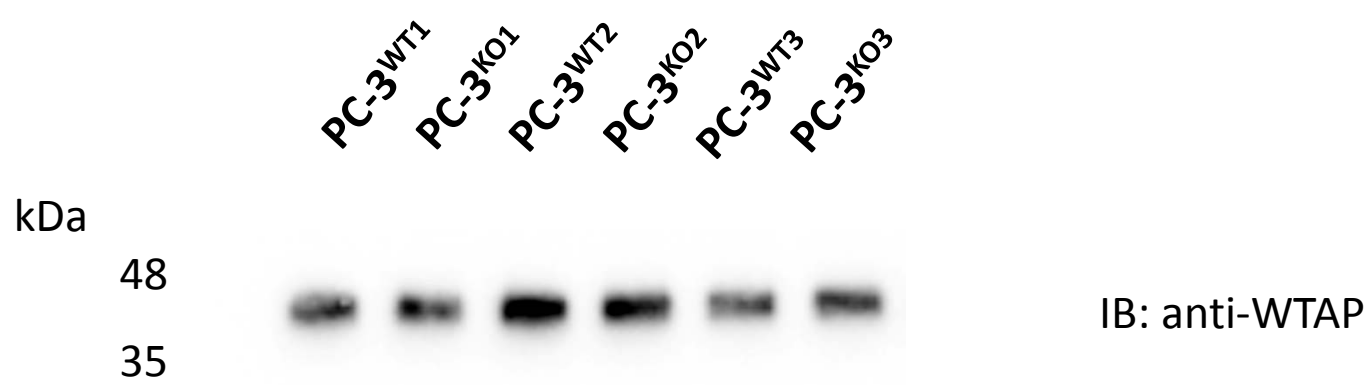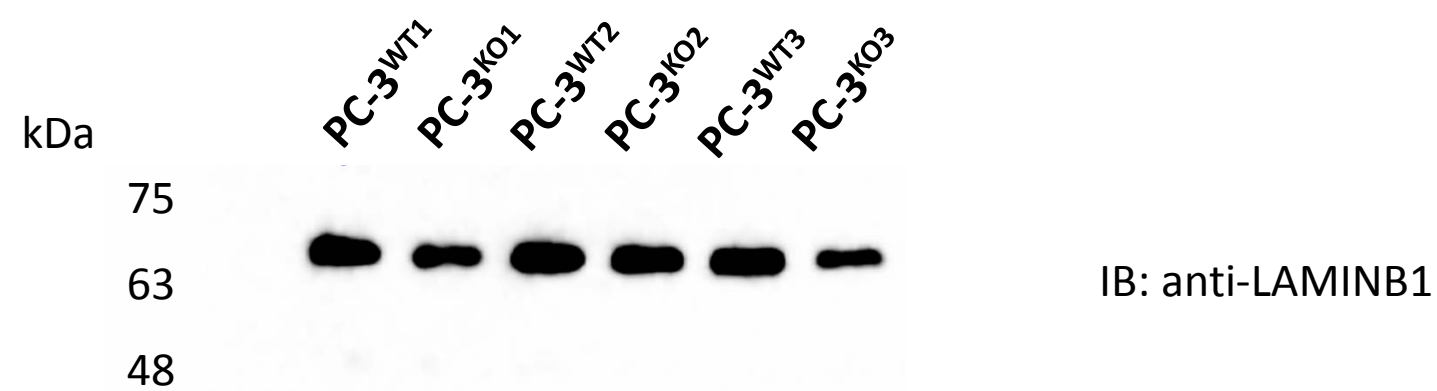

Supplement: Supplementary file 1 [file cancers-12-00771-s001.zip › Supplementary Files/Figure S1.pdf]
